# Towards sustainable emergence transportation system for maternal and new born: Lessons from the m-mama innovative pilot program in Shinyanga, Tanzania

**DOI:** 10.1371/journal.pgph.0002097

**Published:** 2023-06-21

**Authors:** Belinda J. Njiro, Jackline E. Ngowi, Linda Mlunde, Castory Munishi, Ntuli Kapologwe, James T. Kengia, Linda Deng, Alice Timbrell, Wilson J. Kitinya, Bruno F. Sunguya

**Affiliations:** 1 School of Public Health and Social Sciences, Muhimbili University of Health and Allied Sciences, Dar es Salaam, Tanzania; 2 President’s Office Regional Administration and Local Government, Dodoma, Tanzania; 3 Touch Foundation, Mwanza, Tanzania; Asian University for Women, BANGLADESH

## Abstract

Maternal mortality comprises about 10% of all deaths among women of reproductive age (15–49 years). More than 90% of such deaths occur in low- and middle-income countries (LMIC). In this study, we aimed to document lessons learnt and best practices toward sustainability of the m-mama program for reducing maternal and newborn mortality in Tanzania. We conducted a qualitative study from February to March 2022 in Kahama and Kishapu district councils of Shinyanga region. A total of 20 Key Informant Interviews (KII) and four Focused Group Discussions (FGDs) were conducted among key stakeholders. The participants included implementing partners and beneficiaries, Community Care groups (CCGs) facilitators, health facility staff, drivers and dispatchers. We gathered data on their experience with the program, services offered, and recommendations to improve program sustainability. We based the discussion of our findings on the integrated sustainability framework (ISF). Thematic analysis was conducted to summarize the results. To ensure the sustainability of the program, these were recommended. First, active involvement of the government to complement community efforts, through the provision and maintenance of resources including a timely and inclusive budget, dedicated staff, infrastructure development and maintenance. Secondly, support from different stakeholders through a well-coordinated partnership with the government and local facilities. Third, continued capacity building for implementers, health care workers (HCWs) and community health workers (CHWs) and community awareness to increase program trust and services utilization. Dissemination and sharing of evidence and lesson learnt from successful program activities and close monitoring of implemented activities is necessary to ensure smooth, well-coordinated delivery of proposed strategies. Considering the temporality of the external funding, for successful implementation of the program, we propose a package of three key actions; first, strengthening government ownership and engagement at an earlier stage, secondly, promoting community awareness and commitment and lastly, maintaining a well-coordinated multi-stakeholder’ involvement during program implementation.

## Introduction

Maternal and neonatal mortality remains a global health problem [1]. Despite the efforts to address maternal mortality, it is the leading cause of mortality among women of reproductive age (15–49 years) in sub-Saharan Africa, accounting for 10% of all deaths in this age group [2, 3]. Globally, Ninety-four percent of maternal mortality occur in low- and middle-income countries (LMIC) and about two third (66%) of these occur in the sub-Saharan Africa [4]. This is against the backdrop of poor reporting of a significant number of maternal deaths in developing countries which may go uncounted and some dying before they reach health facilities [5].

Maternal mortality is linked with the three-delay model proposed by Thaddeus and Maine [6]. The first delay occurs at the community or family level. It is the delayed decision to seek care. This can be caused by a lack of knowledge on the emergency signs, a lack of funds to facilitate transport to health facility or restricted decision making for women at a family level. The second delay is associated with poor accessibility of health care. It is the delay in reaching health facilities which can be caused by lack of timely transportation system or poor infrastructures. The third delay is in receiving adequate and appropriate care at the facility. This has been attributed to delayed referral, lack of essential drugs and lack of adequately trained health personnel [7, 8].

Improving maternal health is one of the sustainable development goals (SDGs). Specifically, it is target number 3.1 (Ending preventable maternal mortality) with the aim of reducing maternal mortality ratio (MMR) to below 70 deaths per 100,000 live births in 2030 [4]. Expedited efforts need to be taken to achieve this goal. This can be done through Integrated interventions to address the three delays. In Tanzania, all three delays play a role in maternal and neonatal mortality with the third delay contributing to most of the reported maternal mortality [9]. Proposed evidence-based interventions include promoting birth preparedness and community-based education, advocacy and key messages on danger signs [10]. Addressing barriers to reaching care through supporting emergency transportation and improving infrastructures for health [11], is another intervention. Addressing the third delay through improving facility supplies, standard trainings and supervisions for health personnel [12, 13] is the other intervention.

With much efforts invested into implementing evidence-based interventions in LMICs, most of which are externally funded, it is essential to ensure that such programs are long lived and the community continues to benefit beyond initial Implementation [14]. Sustainability has been defined as ‘the degree to which an intervention continued to be implemented after initial effort to secure adoption is completed’ [15]. An intervention is considered sustainable if the intended services are institutionalized and continue to be delivered over time after the external support is terminated [16]. Sustainability of any effective intervention is crucial to ensure continuation of the observed results. A program that does not have the ability to maintain its activities at capacity is prone to be wasteful and distort community trust in the process [17].

Tanzania has witnessed a steady decline in neonatal and child mortality, but not maternal mortality [18]. Counterintuitively, in 2015, MMR was reported at 432 deaths per 100,000 livebirths with the trend of MMR decline is reported at 2.2% per year [18]. However, maternal mortality burden was unevenly distributed with some regions experiencing even a higher maternal mortality than the national average, Shinyanga being one of them with a MMR of 446 deaths per 100,000 livebirths [19]. Most maternal deaths are attributed to direct causes such as hemorrhage, infections, pre-eclampsia, obstructed labor and unsafe abortion [8]. To reduce maternal and neonatal morbidity and mortality in Shinyanga, the m-mama program was implemented through strengthening the health systems, addressing the three delays, and improving the capacity of health workers. The evaluation of m-mama interventions showed the program successfully contributed to the reduction of maternal and neonatal mortality in Shinyanga. However, it is not known how the observed results can be maintained and institutionalized in both rural and urban areas in Tanzania and other countries with similar contexts. We highlighted the stakeholders’ and beneficiaries’ perspectives on the sustainability of the m-mama program for the reduction of maternal and neonatal mortality in Shinyanga. This study therefore aimed to assess the sustainability of the m-mama program outcomes based on the government’s commitment and functioning of the embedded processes so far.

## Methods

### Study design and setting

A qualitative study was conducted between February and March 2022 in Kahama and Kishapu districts of Shinyanga region, Northern Tanzania. Kahama and Kishapu are among the 6 municipalities in Shinyanga where m-mama program was implemented. In 2015, Shinyanga was reported as one of the regions with higher MMR of 446 deaths per 100,000 live births than the national average of 432/100,000 live births [19].

### Description of the m-mama program in Tanzania

The m-mama program was implemented in the Mwanza and Shinyanga regions in Tanzania in various phases. The program began in 2013 as the Mobilizing Maternal Health project. It aimed to address broad, systemic challenges through a coordinated horizontal program approach that extends from the community, lower-level health facilities up to the hospital level. It has been implemented in close collaboration with government partners at the Ministry of Health (MoH) and the President’s Office Regional Administration and Local Government (PO-RALG), selected health facility governing bodies, and communities. The program focused on proof of concept and identifying cost effective solutions to address maternal and neonatal mortality. In the current phase, the program started in 2017 to expand m-mama program into the entire Shinyanga region with a total of six councils and approximately 1.9 million population. It focused on the ownership and sustainability of the program by local government authorities beyond the donor supported services. Touch Foundation, Pathfinder International and respective Regional and Council Health Management Teams (R/CHMT) were the key implementers of the m-mama program.

### m-mama program interventions

m-mama program interventions included the Emergency transportation system (EmTS) and technical and operational support of the health system. The EmTs was designed in concert with the Ministry of Health (MoH), Reproductive and Child Health Services (RCHS) division and the Regional Health Management Team (RHMT). This innovative method utilized technology to remotely triage patients and dispatch an ambulance or community taxi driver to transfer the patient to a health facility. It aimed to address the second and third delays (minimizing delays in reaching health facilities and delays in receiving appropriate health care).

With the aim of providing holistic care for women and newborns, m-mama program also provided technical and operational support. This included the establishment of Community Care Groups (CCGs) to address the first delay by influencing proper health-seeking behaviors in the community. Also, the program supported health facilities in Shinyanga region to improve the quality of Maternal, Neonatal and Child Health (MNCH) care at Basic Emergency Obstetric and Neonatal Care (BEmONC) dispensaries, health centers and hospital level by building the capacity of health providers, providing equipment and improving detection and referral of high-risk and complicated pregnancies. This assured provision of high-quality care for women and newborns upon reaching the appropriate health facility. Concurrently, Touch Foundation helped to build clinical and structural capacity at the health centers and district hospital level for the provision of Comprehensive Emergency Obstetric and Neonatal Care (CEmONC) services so that together the activities supported a continuum of care for women to access MNCH services at all levels of the health care system.

### Conceptual framework

We adopted the integrated sustainability framework (ISF) inductively to synthesize themes and subthemes on program sustainability. With the ISF, the important constructs listed as factors influencing program sustainability across settings are the outer context, inner context, the processes, characteristics of the interventionists, and characteristics of the intervention [20]. The ISF establishes that sustainability should be conceptualized as a process rather than an outcome, expanding on the dynamic nature of sustainability. Further, ISF adds on the previous framework that considers sustainability as sole function of interventions’ characteristics [21] or contextual factors [22]. For this matter, this framework considers inclusivity of both interventions’ characteristics and contextual factors as crucial determinants and ingredients to program sustainment. ISF has been used in other settings to evaluate the sustainability of evidence-based practice in understanding facilitators and barriers to program sustainment [23].

**Fig 1:**
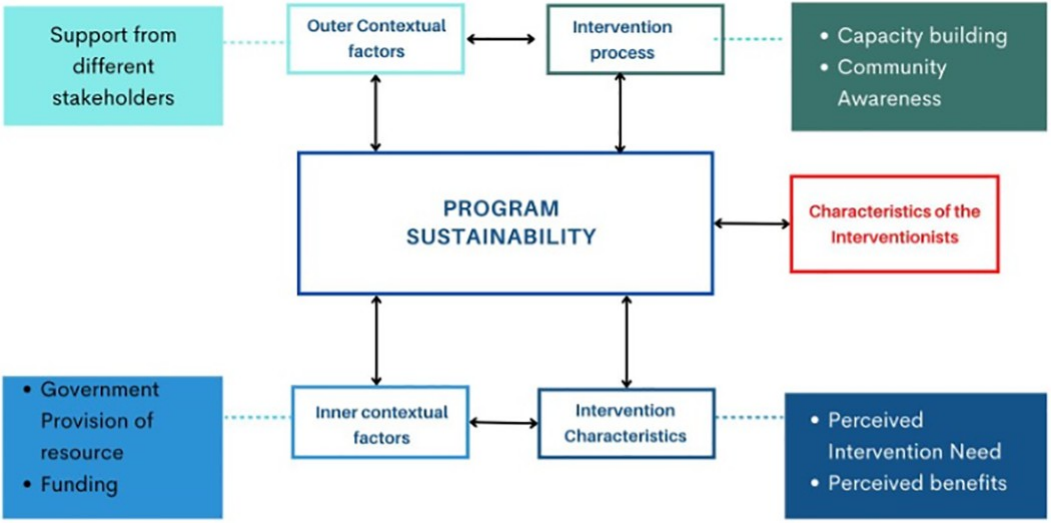
Major themes depicting factors associated with the m-mama program sustainability in Shinyanga, Tanzania. Adopted from the Integrated Sustainability Framework (ISF) [20].

The themes selected and developed in our study were analysed and interpreted using the domains of the ISF in the dynamic context as shown in Fig 1. We utilized the articulation and interaction of the four major domains included in the interventions and contextual factors to analyse and provide evidence on the dynamic roles of sustainability using the documented stakeholders’ and beneficiaries’ perspectives. The constructs included are the outer context (*i*.*e*., support from different stakeholders as significant contributor in supporting program continuation), the inner contextual factors (*i*.*e*., government commitment through provision and maintenance of resources), the intervention characteristics (*i*.*e*., perceived need and benefit of the intervention) and the intervention process (*i*.*e*., continued capacity building, community awareness and close monitoring of the implemented activities).

### Study participants

Respondents in the current evaluation study categorized in five groups. The first group included government officials at national, regional and district levels. At national level, stakeholders from PO-RALG and Safe motherhood from the MoH were interviewed. Also, government officials, coordinators and officers at regional and district level were included.

Program staff from m-mama, Pathfinder and Touch Foundation at administerial level as well as m-mama Program implementers form the second group of study participants. These are; District Transport Officers, Emergency Transport System Focal persons and Dispatch supervisors. Thirdly, at community level, we involved Community Care Group (CCGs) facilitators responsible in facilitating and supervising CCGs. The fourth group were the program implementing partners at the local facilities and communities, these are the health care workers (nurses and doctors) at health facility level, and drivers and dispatchers included to assess the emergency transportation system employed in improving health facility accessibility. The last group included the beneficiaries of the m-mama program, these were selected women or mothers at community level and their spouses.

### Recruitment of study participants

Purposive sampling was conducted from the level of region to health facility. Purposive sampling is commonly and widely used in qualitative research to obtain information-rich individuals to ensure in-depth understanding of the subject of interest [24]. It involves selecting research participants according to the needs of the study and researchers choose participants (individuals, groups or organisations) that have particular qualities or characteristics of interest.

At the regional level, two of the six councils covered by the program were purposively selected to be included in the evaluation; Kahama MC and Kishapu DC were the selected councils. Then, at the council level, one health facility was selected purposively from each council. Staff from selected health facilities were recruited through support from the district council. CCG facilitators and supervisors, drivers, dispatchers and beneficiaries were recruited both at the health facilities and in the community with the support of staff from the health facilities.

### Data collection methods

A total of four (4) experienced research assistants fluent in English and Swahili languages were recruited and trained by senior researchers involved in this evaluation on the qualitative study design, interviewing techniques and type of data to be collected. One day was used to pre-test the tools as part of training and validation of the tool. They were also oriented on the ethical considerations pertinent to this study. Interview guides were developed using extensive review of the interventional activities during the program implementation and review of literature on sustainability. The major sustainability questions are reported in Table 1.

**Table 1:** Evaluation questions to assess program sustainability at all levels.

Local government ownership	Sustainability
How has the RHMT/CHMT been involved with the implementation of the emergency referral system? What has worked well? What were the challenges in maintaining program activities?	In what ways has the RHMT/CHMT been supporting the transport system after introducing community transport systems? In what ways can the emergency transport system continue functioning/operating and be maintained without donor support? What has worked well in the partnership between government and implementing partners in sustaining the referral systems? What has been challenges and how we can improve the partnership in the next phase to ensure program sustainability? What should be done to ensure sustainability of the program?

We used two qualitative data collection methods, 38 Key Informant Interviews (KII) were conducted among key stakeholders, implementing partners and beneficiaries. Seven (7) Focused Group Discussions (FGDs) were completed for CCG facilitators, facility staff, drivers and dispatchers to gather data on their experience with the program, services offered, and recommendations to improve sustainability of the services implemented under m-mama program. On average, six (6) participants were included in each FDGs. Research assistants had no prior relationship with all the interviewees; before starting KIIs and FDGs, researchers used a few minutes for introductions to ensure rapport and trust from the participants. All KIIs and FDGs were conducted considering participants’ conveniences and preferences. For health facility staff and dispatchers, FDGs were conducted at a selected venue in the respective facilities. Similarly, FDGs with drivers and CCGs were conducted in the health facilities where m-mama program was implemented. For key stakeholders and implementing partners, we conducted the KIIs in their respective offices. On average, the interviews lasted for 45 to 60 minutes.

### Data management and analysis

All the KIIs and FDGs were conducted in either English or Swahili and they were audio-tape recorded after consent was sought. KIIs and FDGs were transcribed verbatim in Swahili and in English and translation was done for data collected in Swahili. Transcriptions were done immediately within 24 hours to allow for any clarifications and to assess data saturation. Two members of the research team reviewed the transcripts interactively and inductively determined themes and subthemes. Thematic analysis was used as highlighted in five stages; data familiarization, generating initial codes, determining themes from codes, reviewing themes and lastly results presentation [25]. For illustration purposes, key quotes were selected for all themes and subthemes.

### Quality assurance

Arrangements were made throughout the research process to ensure maintenance of the standards of conducting qualitative research as reported elsewhere [26]. During the study design, investigators conducted a desk review of the m-mama program progress reports, monitoring and evaluation reports and reviewed similar research to generate a comprehensive data collection tool and assure inclusivity and capturing of all relevant aspects of sustainability.

All research assistants were selected based on the experience in conducting interviews in our setting. A three-day training was conducted for research assistants and one day used for the pretesting and validating of tools. All KIIs and FDGs were audio-recorded to ensure that all the information is captured. Transcriptions were done simultaneously and reviewed by at least one investigator daily to check for quality. During data analysis, transcripts were read repetitively and emerging themes recorded in a codebook. Two investigators conducted data coding and one experienced colleague reviewed the codebook. All the transcripts and codebook were stored in an encrypted file which only investigators had access to.

### Ethical consideration

Ethical approval for this evaluation was granted by the Muhimbili University of Health and Allied Sciences Research and Ethics Committee. Permission to collect data from Shinyanga region was granted by the President’s Office Regional and Local Government Authority and the office of the Regional Medical Officer in Shinyanga. Written informed consents to audio-record and participation in the study were obtained from participants before data collection. Participants were given space to decide about their participation. They were also informed that their participation is voluntary and they may wish to withdraw their participation at any point. Privacy and confidentiality were maintained. The data obtained from this program evaluation were kept as strictly confidential, were accessible to only the named investigators and has been stored on password-protected computers.

## Results

### Participants characteristics

A total of 80 participants were enrolled in this study; Of these, thirty-eight 38 (participated in KII and 42 in the seven FDGs. Participants’ mean age was about 38 years and slightly above were males. Program officials and government officers (n = 14, 17.50%) were involved in administrative works at district, regional or national levels. (Table 2)

**Table 2:** Demographic characteristics of the stakeholders interviewed (N = 80).

Variable	Categories	n (%)
**Interviewee group**	Health facility staff	15 (18.75)
	Program / Government Officials*	14 (17.50)
	Community workers / facilitators	18 (22.50)
	Dispatchers	19 (23.75)
	Drivers	9 (11.25)
	Beneficiries / Parents	5 (6.25)
**Sex**	Male	42 (52.50)
	Female	38 (47.50)
**Mean Age (Range)**		38.32 (26–59)
**Professions / Roles**	Government / Program officials	14 (17.50)
	Medical doctors / Clinical officers	6 (7.50)
	Nurses	35 (43.75)
	Drivers	9 (11.25)
	Community workers / facilitators	16 (20.00)
	Volunteers / Others	14 (17.50)

*Participants were merged to ensure confidentiality

### Best practices and lessons on facilitating sustainability of the m-mama program

The factors for promoting sustainability of m-mama program reported by different stakeholders are documented in the four domains of the ISF. *Support from different stakeholders as significant contributor in supporting program continuation* was reported in the outer context. Related to the inner context is the *government commitment through provision and maintenance of resources*. Regarding the processes involved in the implementation of the intervention, *continued capacity building and community awareness* was suggested, as well as close monitoring of the implemented activities to ensure smooth and well-coordinated delivery of the proposed strategies. *The perceived need and benefit of the intervention* is included in the intervention characteristics domain (Fig 1). However, not much was reported on the characteristics of the interventionists as one of the domains of ISF.

#### Government commitment through provision and maintenance of resources

The government involvement from the beginning of the program implementation was seen to be key to ensure program sustainability. The government through the district councils were not only considered as the key drivers of implementation but also as the main catalyst for influencing the ownership and continuation of the m-mama program initiatives at the local levels; as one of the stakeholders stated;

*“Government engagement from the beginning is important*, *it was very clear*, *we did handover training on how to train the dispatchers*, *capacity building for the government teams*, *it makes the government feel the ownership… Working together to get the best outcome given what we need for the system*. *The government is a part of it*, *they own it”*

*- stakeholder*, Touch Foundation

For the program to be sustained, the government needs to be committed. The government has to make sure that the resources needed to run the program are available by including the program needs in the budgets at every level. For example, councils should have a budget to enable the provision of services introduced by the program. The health system in Shinyanga allows the district councils to develop and report their budgets through the Comprehensive Council health Planning (CCHP) for funding by the government; participants highlighted that the priorities should be placed at the council level to make sure that the program activities are well budgeted.

*“The government should support the review of guideline*. *The CCHP at the councils should capture transport costs*, *if the costs are not captured in the CCHP*, *it will not be recognized by the government*, *this will make the funds available to sustain the program”*

*- stakeholder*, Pathfinder

*“Regarding sustainability*, *as councils we have to include the program in the budget so that we continue to get services from the drivers because they were trained and they are willing to volunteer so that we support each other in reaching our clients”*

*- Medical officer*, Kishapu

Delayed funding from the government affects the sustainability of the program activities. One of the reported concerns was payments of community drivers involved in the emergency transportation.

*“We still use this program*, *we still continue to sustain it but as I told you*, *the challenge is with our financial systems for paying these drivers*, *it becomes difficult at times*, *program sustainability will be inadequate compared to when they were paid by the funders*, *on the spot*. *They were paid as they transfer clients*, *this encouraged them to work*. *Currently*, *we only work with the drivers that are considerate and willing to help but not all of them are patient enough to continue providing services*.*”*

– HCWs FGD, Kishapu

The delay of payments to the drivers causes them to leave the program activities and subsequently creating human resource shortage. This is demonstrated in the quote below:

*“Another thing*, *I don’t know how to tell you this*, *most of the drivers currently have ran away because of the payment issues*. *If the driver transfers the patient*, *they are supposed to be paid by the facility*, *at the facility*, *they will have to wait for the basket funds for them to be paid*.”

- *Dispatcher General*, Kishapu

Most of the community mobilization activities were done by the community health workers (CHWs). Most of these are not regularly paid by the government. The provision of incentives to the CHWs and dispatchers was reported as another initiative towards sustaining the new program interventions especially community-based activities.

*“*…*due to the fact that they thought if we continue with this work*, *we might receive some income*, *some continue*, *and some had to stop*. *In order to help them earn at least an income*, *we can include them in the budget if possible*. *Initially results based financing (RBF) was helpful in this but currently RBF is unpredictable*.*”*

*- Community stakeholder*, Kahama

*“For these NGOs or the government*, *the government should see the role of including them in the monthly budget*, *those community-based workers*, *because they are doing volunteering work up to today*. *Since this is very important*, *lifesaving and it raises the national economy*, *if the mortality and morbidity are reduced*, *these people would be contributing in productive works so they should remember the people supporting the women…”*

- FDG, Kahama

The m-mama emergency transportation program was mainly implemented through the available ambulances at the facilities and the community taxes. However, the ambulances at the facilities needed some renovations. The need to increase the number of ambulances was suggested by one of the stakeholders. It was argued that depending on community taxies alone is risky because they can be unpredictable.

*“For this program to be sustained*, *the major thing is to increase the number of ambulances*, *I think ambulances are more important*. *Community tax drivers have other responsibilities*, *they have signed the contract but they have other responsibilities as humans*. *They may fall sick*, *or travel e*.*t*.*c*, *but with the ambulances in many facilities*, *it helps with the facility activities*. *Even if I go to holiday*, *someone else will keep working so it is more sustainable*. *It is different from relying on the community tax drives only*. *To make it easy*, *they should bring more ambulances in the facilities*.*”*

*–Medical Officer*, Kahama

The government is called to improve the infrastructure especially roads. Good roads will enable smooth operation of the emergency transportation. Also, they will enable the transportation of medical supplies to the health facilities so that patients can get the services they need on time.

*“For the councils near me*, *for instance*, *Msalala and Ushetu*, *infrastructures are still a big challenge especially for small cars*, *there was a day a car got stuck and the patient was delayed for about 8 hours and it had to be dragged*. *You might transfer the patient experiencing complications and you find the roads are bad and you get stuck… the infrastructures are not friendly at all…”*

- Officer, Kahama

To curb the challenge of poor transport infrastructures, it was suggested that the government considers increasing the number of CEMONC centers so that reaching the health centers take a considerably short time.

*“For what I think*, *improving the infrastructures with the way our district is*, *it may take a long time*, *but more CEMONC centers should be built*, *so that we reduce the distance*, *this is one of the ways to solve the problem”*

–*CCG facilitator*, Kishapu

#### Support from different stakeholders played a significant role in supporting program continuation

Throughout the implementation of the program and towards the end of program, the m-mama program team trained and involved the respective councils to promote the ownership of the program and government preparedness to take full responsibility of the program. However, participants thought that the donors are still needed for the program to achieve the desired impact and ensure sustainability.

*"With regards to EmTS*, *if stakeholders would support*, *it means the project would be going on well*, *but for them to drop and leave the health facilities*, *it was too early*. *Therefore*, *they should continue to handle it because the government has many things to do*. *We should not say that the responsibilities of the project go back to the government*. *They have shown the way on what has to be done but sustainability is a challenge*,*”*

- *CCG facilitator*, Kishapu

Regular program monitoring from the m-mama supporters and innovators was suggested so that the government continues to learn from the innovators and m-mama stakeholders. It was also perceived that the m-mama program is “their project, they started it” so it is crucial that they continue supporting it.

*“For what I see*, *what is needed is continued support*, *even if the donors’ contract has ended*, *they are supposed to continue supporting because this is their projects*, *they started it*. *So*, *they should continue for some time to see how the program progresses while receiving support from the government too… Even now the government has taken over*, *but we had more drivers showing up initially when the donors were still supporting*. *They should observe what causes the drivers to exit the program*, *they should implement what the donors used to do and maintain it so that the program is sustained*.*”*

*- Facility Based Coordinator*, *Kahama*

The government resources were seen as inadequate to ensure sustainability of the program, a call was made for other stakeholders to supplement what is implemented by the government.

*“… For instance*, *for what I see*, *the services are ongoing but to a low standard because initially*, *the government was working with support from the stakeholders*, *for instance the referral system was there before*, *and even now it is there*, *but extent of service delivery is low because the high cost might overburden the system*. *For instance*, *the ambulances were very few so the funder introduces community taxes; so if the government fails to pay the tax drivers*, *then these services will not be provided adequately and we will end up depending on ambulances only*. *We might go back to how it was in the past before the program*. *The stakeholder’s involvement will really help because now the program is preforming poorly*.*”*

–FDG, Kishapu

Capacity building as one of the core program activities provided during implementation of the m-mama program played a role in reducing maternal and neonatal deaths. This learning environment supported by the funders and stakeholders was seen as an initiative towards better maternal and child health outcomes.

*"We wish that the project is brought back and continues to support us*. *It helped us to provide education to the community*, *and community members have started to understand what is supposed to be done*. *So*, *if they continue to support through this project*, *to reduce maternal and neonatal deaths*, *save lives of mothers and children*, *we will be happier*,*”*

- a Community Based Coordinator reported.

Not only the involvement of multiple stakeholders is needed but also a well-coordinated partnership between the government and these stakeholders to ensure proper planning and utilization of resources

*“The government is the key player in the Ministry of Health*, *it is stakeholders’ responsibility to see where the government has limited ability so that they can proceed from there*. *I don’t think it’s wise if the government provides gloves and the program also provides gloves (laughing)*, *there will be overstock and it won’t have much impact*. *The important thing here is observing where the government efforts have ended so that the stakeholdesr can complement”*

- *Medical Officer*, Kahama

However, others thought that the government is swamped with many responsibilities, therefore the donors should continue to support the program so that the community can continue to enjoy its success and benefits.

*“Maybe if they can use the method they used initially*, *because it was Touch and pathfinder*, *they were good*, *they were the ones paying the drivers for a significant proportion and the drivers were paid timely*. *If they can improve and revert to how it was*, *then all will be well*.*”*

–*hospital attendant*, Kishapu

*“So*, *what to consider is… if the program is progressing well*, *they should strive to handle it themselves*, *the government has so much to do*, *we shouldn’t leave this to the government*. *Of course*, *they have shed some light on what is supposed to be done and how it should be done*, *but based only on that*, *sustainability will be low*.*”*

- *CCG facilitator*, Kishapu

#### Continued capacity building and community awareness

To continuously reap the benefits of this program, health care workers advised on the presence of regular capacity building and refresher trainings to ensure the possibility of responding to challenges faced during program implementation timely and efficiently.

*“When it comes to training*, *I think they have done enough and we have understood*. *But there should be regular*, *short term refreshment courses to discuss on solving the challenges we meet*, *it shouldn’t take so long like 6 months but it can be every two months to evaluate on the challenges faced and address them timely*.*”*

*–dispatcher*, Kahama

Regarding the trainings that were primarily done by the government, the commitment to make the curriculums more practical was suggested. The trainings are inherent responsibilities of the government and the respective councils; the m-mama program promoted timely and regular conduction of these trainings but the role lies inherently on the respective councils as stated;

*“This is more of the government commitment; it is not a project issue*. *The government needs to change the curriculum for BEmONC*, *CEmONC and Neonatal Intensive care to be more practical rather than theory classes*. *This is championed by the MoH*.*”*

*- stakeholder*, Pathfinder

Raising awareness of the community can contribute in stimulating the utilization of the program activities; this is crucial to ensure that the program is sustained. The community care groups led by the CHWs were the most utilized platforms for raising awareness among expecting mothers and encouraging proper practices towards safe delivery.

*“… Our community needs to be educated adequately*, *some of the things we observe are due to lack of education*, *when you provide education at community level*, *the fruits and benefits are great as I said earlier…*.*”*

*- CHW*, Kahama

Since the CCGs were led by the members of the community, they were perceived to be easily sustainable.

*“In forming these community groups*, *we used community care group facilitators*, *these are known in the particular societies in engaging and mobilizing their communities well… This was intentionally done so that sustainability is feasible*, *it is the community members themselves making the initiative to have such groups*, *we therefore expect that the knowledge is retained in the community and they maintain the services*.*”*

*- stakeholder*, Pathfinder

*“Also empowering the community has been a great success in raising awareness*, *women are now referring themselves through the community care groups”*

*- stakeholder*, Touch Foundation

To ensure that all community members are covered by the program, it was suggested that the media platforms such as local radios be used to educate the community on the maternal and newborn health services provided.

*“We can do advocacy*, *we may use the local radios*, *we can have sessions*, *health education*, *we can also organize community campaigns to educate the society on the presentation and what to do if they see such and such signs*. *So*, *both these things will help in informing the community on what to do in case of emergencies*.*”*

*–medical officer*, Kahama

Community awareness to increase trust of the program and ultimately maintain the utilization of the services even without funders involved is crucial.

*“But the community should cooperate and continue calling and utilizing the services*. *We usually have the perception that the program can only succeed if it is a white person leading it*, *but if it’s just African*, *people start to think nothing will work*. *So*, *they shouldn’t stop using this program*, *they should continue calling*, *we will continue serving them for 24 hours*, *seven days a week*. *The community leaders should keep helping us…”*

*- Transport Focal Person*, *Shinyanga MC*

The m-mama program provided a toll-free contact number available 24 hours for reporting on maternal or neonatal emergencies. It was suggested that efforts on the provision of continuous community awareness should also include reminders on the toll-free numbers as other villagers may not know these numbers.

*“Also*, *to improve and strengthen this program*, *education should be provided regularly in the community*, *we might assume that the toll-free number have been distributed but other people have never received them so it is important that we educate them frequently after every few months in the villages*. *People are informed and given the toll-free number…”*

–HCWs FDGs, Kishapu

Everyone in the community should be aware and involved in the emergency transportation system. A call was made for the community leaders to be directly involved in increasing the visibility of the program and promote its utilization through the local platforms such as annual meetings.

*“The community leaders should continue helping us*. *We still go back*, *asking them to encourage their societies in the community or government meetings*, *one of the agenda there should be to inform them that this program is to save the lives of mothers and newborns*. *Despite the donors exiting*, *the government has taken over and it is implementing it a hundred percent*.*”*

- Transport Focal Person, Kahama MC

Male involvement is another area that was reported to play a key role in services utilization by the community. Community awareness activities were emphasized to be inclusive of males as they are the “main decision makers” in the families.

*“We should find a way to make men feel as they are part of the program*, *because they used to think it is only for women and newborns*, *this brought up challenges… If we could then educate them too and not only women so that they are also involved*, *to include them even in the community groups*. *They used to say those are just for women*, *and their stuff*, *we should involve them because they are the main decision makers”*

–CCG facilitator, Kishapu

#### Dissemination and sharing of evidence and lesson learnt from successful programs

The need to continuously gather evidence throughout the program and share the impact with the decision makers was suggested so that people in the central government can understand what is happening and share the story in high level meetings to get a policy buy-in.

*“Another lesson learned is that collaboration with the government is key*, *a key success factor*, *their enthusiasm is key to success*, *getting their buy in*, *that this isn’t something that only we are doing*, *we are doing it together”*

– Program manager, Touch foundation

*“In record keeping*, *the data that we keep*, *I know there are challenges*. *If we maintain those data well*, *others can visit and see what we do and learn through such data*. *This will help to showcase how this program helps a lot of people*.*”*

*- dispatcher*, *Kishapu*

All service providers should be informed of the program and its contents. Orientation of the new staff employed in the facilities is crucial to ensure the continuity of care for patients and introduction of the program for those not in the cycle.

*“For instance*, *I can say in the facility*, *if we get extra workers*, *we usually organize a meeting*, *we orient them on what will be going on so that if they are the ones on duty at the facility*, *they can provide services*. *When they come*, *we inform them on the ongoing support and organizations we work with…”*

–HCWs FDG, Kishapu

Having actual evidence through gathered data and regular program outcomes monitoring is not only essential for sustainability but may also promote program scale up.

*“We need to keep gathering evidence*, *we have communicated with the ministry and there is an agreement for us to expand into Lindi and Morogoro and have agreed for us to expand to 14 other regions*. *Direct financing did not come overnight*, *you need to gather evidence for them to see*. *We need to be do doing mid-term evaluations*, *if we don’t gather evidence well*, *we will fail to convince people*”

–stakeholder, PORALG

#### Close monitoring of the implemented activities to ensure smooth and well-coordinated delivery of the proposed strategies

The role of monitoring and evaluation was perceived as a paramount continuous process that will assist government and implementers to document lessons for future reference. This can be done through continuous learning and improving from the identified gaps, taking note of the lessons learnt throughout the process

*“Aah*, *we need to stick to what we have now and maintain them*, *also to improve when we see there are gaps*. *Also*, *after implementing such interventions*, *to monitor on the remaining challenges and see how we can tackle them so that we can learn and implement what we have*.*”*

–medical officer, Kahama

## Discussion

The current study aimed to document lessons learnt and best practices toward sustainability of the m-mama innovative program for reducing maternal and newborn mortality in Tanzania. We report the following as factors perceived to promote the sustainability of the program by various stakeholders and beneficiaries of the m-mama program. First is the active involvement of the government through the provision and maintenance of resources including a timely and inclusive budget and infrastructure development and maintenance. Secondly, the support from different stakeholders through a well-coordinated partnership. The third factor is continued capacity building for implementers, health care workers and CHWs and community awareness to increase program trust and boost utilization. Then fourthly, striving to disseminate and share evidence and lesson learnt from such successful program activities and lastly, close monitoring of implemented activities to ensure smooth and well-coordinated delivery of proposed program strategies.

Active involvement of the government through provision and maintenance of resources including a timely and inclusive budget and infrastructure development and maintenance is crucial. Program support and leadership from the inner system is a key construct and factor towards sustainability [20]. Program support and trust from the government as the current main implementer of the program was perceived as contributor towards maintenance of the program activities in health care. The government with its internal and external political systems and environment were shown to influence the continuation of the program especially in countries where the main source of funding for health comes from the state [27]. The commitment and political will to accept the ownership of such programs through priority setting and supportive policies have proven to influence the institutionalization process for new interventions in other LMICs [28]. The government’s inability to support health interventions due to financial constraints especially in LMICs have resulted in the termination of life-saving health programs [29].

Although sustainability is a multistage and multilevel approach, the emphasis is on the government for coordination, relevant policy and provision of resources [20]. The current study reports the challenge faced by the implementers at the facility level on delayed funding from the government; this is similar to the Nigerian context where the quick and timely release of budget by the government was addressed as a key action for maternal and newborn health program sustainability [14]. The key intervention in the current proposed m-mama program involves collaboration between the government and community taxi drivers in facilitating timely transportation for all women requiring emergency care; challenges with budgeting and delays are a hindrance to effective and continued delivery of these services especially when external collaborations are involved [14].

Participants in this study highlighted the role of involving other stakeholders in attaining the program goal; specifically, a well-coordinated partnership between the government, donors and the community at large. This finding is consistent with previous studies that report synergies and multisectoral collaborations as facilitators of program implementation and sustainability [15]. Stakeholders’ argument in this study highlighted the government to be inadequate and too swamped to ensure a well sustained m-mama program hence the crucial role of retaining funders and external support. Evidence suggests that independent program implementation by the government beyond external support is possible through full integration of the program activities, building institutional capacity and increasing funding from government sources even during the life of the program [30, 31].

A concept of working within the available resources has been reported in Africa; this assures that the needed framework and funding environment for program implementation is existing hence it is convenient for the program to be sustained even without external funding or support [28, 32]. However, other studies do argue against dependence on donor funds; relying on external funds may affect sustainability particularly because there is an expectation of the donor exiting the program at a later stage which will still require the government’s capacity to take full control. This therefore calls for a long-term program analysis and planning on how the government can independently support such effective interventions [33, 34].

Involving the community in the implementation of program activities was reported as a key role that heightens the uptake of services and promote sustainment. Involving the local community has been shown to promote the quick adaptation of the intervention to local contexts which is crucial for program sustainability [35, 36]. Community engagement has a key role in promoting the accessibility of services by reaching “hard to reach” communities through CHWs and community volunteers [14] and assuring considerations of local needs [37]. Involving the community has also been shown to promote a sense of ownership of the program and empowerment which are paramount in the successful institutionalization of the program [14, 38].

Responses from our findings highlight that some beneficiaries were not well informed about the ongoing interventions for maternal and newborn health such as presence of toll-free number for emergency calls. Some stakeholders suggested increasing community awareness of the program and the role of upholding maternal health through CHWs and the use of media. The CCGs were also the main source of information for most program beneficiaries in our study. Health promotion can also be done through direct community involvement coupled with capacity building as the source of information to recognize emergency signs and symptoms in perinatal period [38].

Regular monitoring of the program activities was perceived as an influencing factor for sustainability. Consistently, continuous reporting of program implementation progress and performance feedback including the challenges during implementation and lessons learnt contribute towards proper program sustainability [39]. Additionally, challenges with local record keeping as reported in our findings pose challenges to sustainability because information on the needed resources and previously used is vital for monitoring and evaluation of the program implementation and future use [36, 39].

The strength of our study is in the use of qualitative method in understanding the value of stakeholders’ perspectives including program beneficiaries on program sustainability. We also built our findings in alignment with the validated integrated sustainability framework. The ISF model integrates a wide range of constructs that are proven to promote program sustainability in a long run. The participants recruited had a deeper and long-standing understanding of the m-mama program hence providing great insight on the what can be done to ensure sustainment. However, our study is not without limitations; Despite the benefit of using purposive sampling, this may have caused selection bias. However, we selected diverse array of stakeholders at all levels of implementation hence allowing triangulation of data in our study. Also, the evidence gathered did not cover all the essential constructs of sustainability on the ISF; however, considering the role of local contexts, some constructs may carry more weight in our context compared to what has been reported elsewhere.

## Conclusion and recommendations

The current study provides context specific qualitative empirical findings in terms of stakeholders’ perspectives on sustaining the m-mama innovative program for maternal and child health. Our findings uphold the crucial role of government preparedness, involvement and support in ensuring the institutionalization of externally funded programs. Stakeholders’ involvement should not stop at the implementation stage but ensure a well coordination partnership during the adaptation stage to promote accountability and adherence to initial implementation plans. We also highlighted the evident role of community mobilization and involvement in program ownership and continuation. Building capacity for HCWs as local program implementers and close monitoring of the program outcomes for future reference and feedback. Given the crucial role of donor funding in implementing health related programs in developing countries, we emphasize on strategies to maintaining programs’ benefits for future projects. We therefore suggest the inclusion of sustainability planning during the design stage and importantly, engaging the community and local government in the design and implementation stages to foster program ownership. Nevertheless, designing self-funding models for emergence transportation through community contributions and involvement of other local stakeholders can be helpful to reduce dependence on the government. Continuing sensitization and raising community awareness on the importance of the designed program in health may assist in demand creation and promoting community engagement and local support for the newly implemented and continuing projects.
